# Long‐term Radiological and Clinical Outcome after Lumbar Spinal Fusion Surgery in Patients with Degenerative Spondylolisthesis: A Prospective 6‐Year Follow‐up Study

**DOI:** 10.1111/os.13350

**Published:** 2022-06-16

**Authors:** Jan Bredow, Carolin Meyer, Stavros Oikonomidis, Constantin Kernich, Nikolaus Kernich, Christoph P. Hofstetter, Vincent J. Heck, Peer Eysel, Tobias Prasse

**Affiliations:** ^1^ Department of Orthopedics and Trauma Surgery, Krankenhaus Porz am Rhein University of Cologne Cologne Germany; ^2^ Center for Spinal Surgery, Helios Klinikum Bonn/Rhein‐Sieg Bonn Germany; ^3^ University of Cologne, Faculty of Medicine and University Hospital Cologne Department of Orthopedics and Trauma Surgery Cologne Germany; ^4^ University of Washington Department of Neurological Surgery Seattle Washington USA

**Keywords:** Clinical outcome, Degenerative spondylolisthesis, Posterior lumbar interbody fusion (PLIF), Radiological outcome, Transforaminal lumbar interbody fusion (TLIF)

## Abstract

**Objective:**

To assess which radiological alignment parameters are associated with a satisfactory long‐term clinical outcome after performing lumbar spinal fusion for treating degenerative spondylolisthesis.

**Methods:**

This single‐center prospective study assessed the relation between radiological alignment parameters measured on standing lateral lumbar spine radiographs and the patient‐reported outcome using four different questionnaires (COMI, EQ‐5D, ODI and VAS) as primary outcome measures (level of evidence: II). The following spinopelvic alignment parameters were used: gliding angle, sacral inclination, anterior displacement, sagittal rotation, lumbar lordosis, sacral slope, pelvic tilt and pelvic incidence. Furthermore, the length of stay and perioperative complications were documented. Only cases from 2013 to 2015 of low‐grade degenerative lumbar spondylolisthesis (Meyerding grades I and II) were considered. The patients underwent open posterior lumbar fusion surgery by pedicle screw instrumentation and cage insertion. The operative technique was either a posterior lumbar interbody fusion (PLIF) or a transforaminal lumbar interbody fusion (TLIF) performed by three different senior orthopedic surgeons. Exclusion criteria were spine fractures, minimally invasive techniques, underlying malignant diseases or acute infections, previous or multisegmental spine surgery as well as preoperative neurologic impairment. Of 89 initially contacted patients, 17 patients were included for data analysis (11 males, six females).

**Results:**

The data of 17 patients after mono‐ or bisegmental lumbar fusion surgery to treat low‐grade lumbar spondylolisthesis and with a follow‐up time of least 72 months were analyzed. The mean age was 66.7 ± 11.3 years. In terms of complications two dural tears and one intraoperative bleeding occurred. The average body mass index (BMI) was 27.6 ± 4.4 kg/m^2^ and the average inpatient length of stay was 12.9 ± 3.8 days (range: 8–21). The long‐term clinical outcome correlated significantly with the change of the pelvic tilt (*r*_s_ = −0.515, *P* < 0.05) and the sagittal rotation (*r*_s_ = −0.545, *P* < 0.05). The sacral slope was significantly associated with the sacral inclination (*r*_s_ = 0.637, *P* < 0.01) and the pelvic incidence (*r*_s_ = 0.500, *P* < 0.05). In addition, the pelvic incidence showed a significant correlation with the pelvic tilt (*r*_s_ = 0.709, *P* < 0.01). The change of the different clinical scores over time also correlated significantly between the different questionnaires.

**Conclusions:**

The surgical modification of the pelvic tilt and the sagittal rotation are the two radiological alignment parameters that can most accurately predict the long‐term clinical outcome after lumbar interbody fusion surgery.

## Graphical abstract

Pre‐ and postoperative (PLIF L4/5) lateral standing radiographs of the lumbar spine are used to measure the pelvic tilt. The greater the difference of the pelvic tilt after fusion surgery compared to the preoperative value, the worse is the outcome after 6 years when quantified by the EQ‐5D questionnaire.

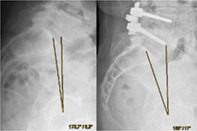

## Introduction

Degenerative spondylolisthesis (DS) is the most common type of spondylolisthesis. The prevalence among women is higher than in men (8.4% and 2.7% respectively). Besides sex, risk factors include race, age above 66 years, obesity, greater‐than‐average height, sagittalization of the facet joints, high lumbar lordosis and high pelvic inclination.^1^, ^2^ In DS, the vertebral body slips forwards or backwards over the adjacent vertebra. In juvenile isthmic spondylolisthesis, a discontinuous pars interarticularis (spondylolysis) causes a vertebral slippage of the two separated parts of the vertebra. Spondylolisthesis can also occur due to pathological osseous changes, dysplastic vertebral arches or after surgery as well as after trauma. Most cases of DS affect the L4/5 level.^1^, ^3^

Displacement of vertebrae can lead to the development of foraminal stenosis and irritation of spinal nerves causing radicular and lower back pain. In 2015, Enyo *et al*. identified a greater risk of progression of DS in female patients younger than 60 years and in patients with facet joint sagittalization.^4^ The study also showed that the progression of DS depends on the extent of initial anterior dislocation of the vertebra and the lumbar axis sacral distance. Lateral radiographs are necessary to diagnose and classify DS. Classification is standardized using Meyerding grades (I to IV) according to the extent of anterior displacement in relation to the adjacent vertebral body. The most severe form is called spondyloptosis and is sometimes considered as Meyerding grade V. In that case, the slipped vertebral body lost contact to the superior endplate of the caudal adjacent vertebra.

In general, low‐grade asymptomatic DS (Meyerding grades I and II) should be treated conservatively. Physical therapy, epidural and transforaminal injections, and oral nonsteroidal anti inflammatory drugs are the first line treatment in patients with symptomatic low‐grade DS. Spontaneous fusion of the affected vertebrae without surgical treatment can also occur over time, which provides an additional reason for initial conservative management.^5^ Higher grades (Meyerding III and IV) of DS, especially in association with chronic lumbar back pain, qualify for a surgical intervention. Regardless of severity, surgical treatments should be thoroughly discussed in patients with progredient chronic or radicular pain and low‐grade DS after exhausting all conservative treatment options.^6^ If neurological symptoms due to DS occur, surgical treatment is capable to restore and maintain neurological function as well as to prevent further progression and loss of sensory, motor, and vegetative functions. Lumbar spinal fusion surgery is a common treatment option for DS in adults. Posterior lumbar interbody fusion (PLIF) and transforaminal lumbar interbody fusion (TLIF) are the most common surgical procedures to treat DS and allow an effective reposition and fixation of the slipped vertebra.^6^, ^7^, ^8^, ^9^, ^10^ Decompression of affected nerve roots and the spinal canal alone can worsen the DS over time by causing instability of the segment. When comparing decompression of the spinal canal by laminectomy only with fusion after the decompression, patients with spinal fusions have better clinical outcomes.^11^ However, when and how to treat low‐grade spondylolisthesis operatively is still controversial and no standardized international management strategy exists so far.^12^

In operative treatment, spinopelvic alignment parameters must always be considered since they play an important role for the development, progression, and outcome of DS. For example, high pelvic incidence, which describes the relation between the center of the femoral head and the S1 plumb line on a lateral radiograph, increases the risk of DS because accordingly compensatory mechanisms need to balance rising shear forces.^13^ Chuang *et al*. compared spinopelvic alignment parameters of patients with and without DS. They identified the sacral slope and the lumbar lordosis to compensate DS best.^14^ Complex activation of muscles, ligaments and joints maintain the ability of standing upright and adapt to any given movement and posture immediately.

In terms of the long‐term outcome, radiographic outcome parameters are not clearly connected with clinical outcome parameters yet.^15^, ^16^, ^17^, ^18^, ^19^, ^20^ Recently published results of 3‐year follow‐up outcome data of patients suffering from degenerative spondylolisthesis who underwent lumbar spinal fusion surgery showed that reduction of the sagittal rotation and the sacral inclination correlates with an improvement of clinical outcome scores collected by the Core Outcome Measure Index (COMI) and the Oswestry Disability Index (ODI).^21^

This study sought to identify and define radiological parameters that are associated with a beneficial long‐term clinical outcome 6 years after surgery. The evaluation of pre‐ and postoperative radiographs of the lumbar spine as well as the development and change of spinopelvic alignment parameters over time can help to gain further understanding of the long‐term influence of fusion surgery in DS. Consequently, spine surgeons would have a guide as to which spinopelvic alignment parameters they should consider achieving an optimal clinical outcome by comparing these parameters with another. The aim of this study was to determine which radiological parameters correlate with a satisfactory clinical long‐term outcome in a cohort of patients that underwent posterior fusion surgery.

## Methods

### Study Design

From April 2013 to December 2015, 89 patients with mild symptomatic degenerative spondylolisthesis (Meyerding grades I and II) underwent open posterior or transforaminal lumbar spinal fusion surgery (PLIF or TLIF). Three different senior orthopedic surgeons performed the procedure, which included fusion of one or two segments. The approach depended on the patient's pathology—in the case of unilateral neuroforaminal stenosis and Meyerding grade I spondylolisthesis, TLIF was preferred. PLIF was performed in cases of multiple neuroforaminal stenosis or grade II spondylolisthesis. Another inclusion criterion was that the surgery was performed at least six years ago. Exclusion criteria were malignant diseases, history of lumbar spine surgery, fusion of three or more levels, spinal (osteoporotic) fractures, acute or chronic infectious diseases, and neurological deficits preoperatively.

The patients' clinical and radiological outcomes were prospectively collected as part of a follow‐up appointment after Institutional Review Board approval (verification code: 09‐182) and after the patients signed the participation agreement.

### Data Collection and Analysis

All data was collected as part of the Spine Tango Register. This included sex, date of birth, body mass index (BMI), operative time, length of stay, and perioperative complications. The follow‐up examinations took place annually after surgery. After collecting the data, it was analyzed using SPSS Statistics (Version 25; IBM, Armonk, NY, USA) by conducting a Student's *t*‐test for paired samples assuming a Gaussian distribution. The Kolmogorov–Smirnov test was used to verify normal distribution for the pre‐ and postoperative data. Correlation analyses of radiological and clinical findings was processed by the Spearman‐Rho bi‐serial test, when the data was not normally distributed. This included the correlation of every single change of each radiological parameter pre‐ and postoperatively with the change of the clinical outcome over time. The level of significance was defined by *P* < 0.05. The statistical evaluation according to Spearman is a correlation coefficient of two ordinally scaled variables. This method shows the strength of a correlation between these two variables. A linear correlation is not assumed when using the Spearman correlation coefficient. The positive or negative correlations are classified into different strengths (“r”) with a range from −1 to +1, defining the maximum of negative (“−1”) and positive (“+1”) correlation.^22^, ^23^

Intra‐ and interobserver reliability were measured by intra‐class correlation. Intraclass correlation coefficient (ICC) values were assessed in a two‐way mixed model with absolute agreement at 95% confidence intervals for interobserver reliability. Values < 0.40 were considered poor, those between 0.40 and 0.59 were considered fair, those from 0.60 to 0.74 were considered good, and those between 0.75 and 1.00 were considered excellent.^24^

### Clinical and Radiological Outcome Measurements

For clinical evaluation questionnaires measuring quality of life including COMI, the European Quality of Life Five Dimensions (EuroQol, EQ‐5D), the ODI and the visual analogue scale (VAS) were used. Different spine societies including the German Spine Society (Deutsche Wirbelsäulengesellschaft, DWG) and the Spine Society of Europe (EUROSPINE) recommend these tests for the quantification of the clinical outcome.

**Fig. 1:**
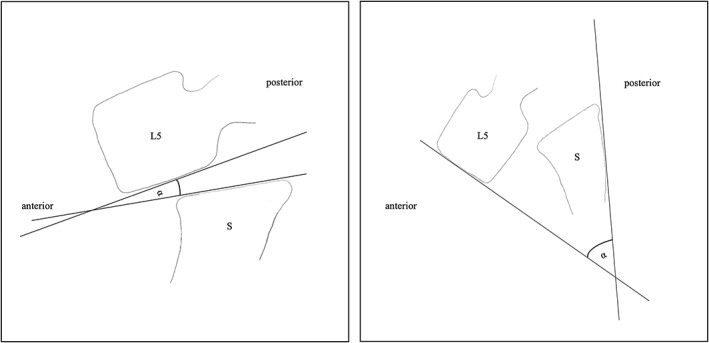
Measurement of the gliding angle (A) and the sagittal rotation (B) in between the fifth lumbar vertebra (L5) and the sacrum (S).

The radiological parameters were measured using standardized conventional radiographs in two planes, one lateral and one front view of the spine of the standing patient. Two different orthopedic surgeons measured the spinopelvic parameters including sacral slope, sacral inclination, pelvic tilt, pelvic incidence, lumbar lordosis, gliding angle, sagittal rotation and the percentage of anterior displacement of the vertebral body as described by Lenz *et al*. and Boxall *et al*.^21^, ^25^ Figure 1 illustrates how the gliding angle, and the sagittal rotation were defined and measured. The grade of spondylolisthesis was defined by the Meyerding classification. Radiographic imaging and completion of the questionnaires were part of the follow‐up examination of this prospectively analyzed cohort.

## Results

### Patient Demographics and Clinical Outcome

Of initially 89 contacted patients, 17 patients replied (19.1%) and were included in this 6‐year follow‐up study (11 males, six females). The average age was 66.7 ± 11.3 years, the average BMI was 27.6 ± 4.4 kgm and the average inpatient length of stay was 12.9 ± 3.8 days (range: 8–21). The 3‐year follow‐up study published earlier included 32 patients, so 15 patients were lost to follow‐up after three additional years. PLIF was performed in 16 of the cases, TLIF in only one case. Of all procedures, 12 patients had mono‐ and five patients had bisegmental fusion surgery. Within the group of monosegmental fusions, the L4/5 segment was fused seven times and the L5/S1 segment was fused five times. The segments L1‐3 and L4‐S1 were fused in one patient each and the segments L2‐4 in three patients. Bisegmental fusion was performed when lumbar segments showed spondylolisthesis and signs of instability as well asassociated osteochondrosis on flexion‐extension radiographs.

The average operative time was 167.6 ± 44.7 minutes with an average blood loss of 707.1 ± 572.5 ml (range: 250 to 2000 ml). Complications occurred in three cases ‐ two of them were dural tears and one was an intraoperative bleeding. None of the patients needed revision surgery.

The results of the different clinical questionnaires among one another all correlated strongly showing significant p‐values (*P* < 0.01).

Six years after surgery, the clinical outcome of the patients was determined by a mean COMI of 4.0 ± 2.84 (range: 0.14–9.57), a mean VAS of 5.4 ± 2.40 (range: 1–10), a mean ODI of 28.94 ± 22.50 (range: 0–72) and a mean EQ‐5D of 0.54 ± 0.35 (range: −0.1–1.0).

COMI and VAS showed a moderately strong negative linear correlation (*r*_s_ = −0.685), COMI and ODI showed high to perfect correlation (*r*_s_ = 0.918) and the COMI and EQ‐5D correlate clearly (*r*_s_ = 0.745) as well. Furthermore, the VAS and the ODI (*r*_s_ = −0.659) and the VAS and the EQ‐5D (*r*_s_ = −0.613) correlate strongly negative. ODI and EQ‐5D correlate strongly as well (*r*_s_ = 0.797). These correlations are supposed to underline that COMI, ODI and VAS all have a high validity in testing the clinical outcome as they are correlating strongly (Table 2).

When looking at the correlations between the clinical and radiological outcomes after 6 years, a change of the mean sagittal rotation from 71.4° ± 4.6 preoperatively to 75.8° ± 11.5 at 72‐month follow‐up exhibits a strongly negative correlation with the ODI (*r*_s_ = −0.545) with a significant p‐value of 0.029. A change of the mean pelvic tilt from 24.0° ± 6.3° preoperatively to 25.1° ± 9.0 at 72‐month follow‐up shows similarly negative correlation with the EQ‐5D (*r*_s_ = −0.515), which is also statistically significant (*p* = 0.041). Only moderate and weak correlations were found regarding the other parameters (see Table 1).

**TABLE 1:** Correlation of the preoperative radiological and clinical outcome measurements with the results 6 years after fusion surgery (significant correlation meaning *P* < 0.05 highlighted by italic (*P* ‐value, “P”) or bold (Spearman's Rho, “*r*_s_”) font)

		COMI	VAS	ODI	EQ‐5D
Gliding angle	r_s_	0.023	0.166	−0.052	−0.040
	*p*	0.929	0.525	0.844	0.880
Sacral inclination	r_s_	0.036	−0.178	−0.043	0.191
	*p*	0.892	0.494	0.870	0.462
Anterior displacement	r_s_	0.255	0.083	0.258	0.028
	*p*	0.323	0.752	0.318	0.914
Sagittal rotation	r_s_	−0.442	0.369	**−0.545**	−0.307
	*p*	0.087	0.159	*0.029*	0.247
Lumbar lordosis	r_s_	0.147	0.035	0.060	0.051
	*p*	0.573	0.895	0.819	0.847
Sacral slope	r_s_	0.047	−0.268	0.020	0.272
	*p*	0.859	0.298	0.940	0.291
Pelvic tilt	r_s_	−0.412	0.321	−0.361	**−0.515**
	*p*	0.113	0.226	0.169	*0.041*
Pelvic incidence	r_s_	−0.138	−0.105	−0.069	−0.108
	*p*	0.610	0.698	0.799	0.690

**TABLE 2:** Correlation of different clinical outcome questionnaires six years after fusion surgery (significant correlation meaning *P* < 0.05 highlighted by italic (p‐value, “P”) or bold (Spearman's Rho, “r_s_”) font) and descriptive statistics of the clinical outcome questionnaires

		COMI	VAS	ODI	EQ‐5D
COMI	r_s_	**1**	**−0.685**	**0.918**	**0.745**
	*p*	*<0.0001*	*0.002*	*<0.0001*	*0.001*
VAS	r_s_	**−0.685**	**1**	**−0.659**	**−0.613**
	*p*	*0.002*	*<0.0001*	*0.004*	*0.009*
ODI	r_s_	**0.918**	**−0.659**	**1**	**0.797**
	*p*	*<0.001*	*0.004*	*<0.0001*	*<0.001*
EQ‐5D	r_s_	**0.745**	**−0.685**	**0.918**	**1**
	*p*	*0.001*	*0.002*	*<0.0001*	*<0.0001*

**TABLE 3:** Preoperative clinical outcome parameters as well as three and six years after fusion surgery treating degenerative spondylolisthesis

	Preoperative	3‐year follow‐up	6‐year follow‐up
COMI	8.2 ± 1.3 (range: 5.5–10.0)	4.3 ± 2.3 (range: 0.0–9.6)	4.0 ± 2.84 (range: 0.14–9.57)
ODI	51.2 ± 19.2 (range: 11.0–98.0)	26.0 ± 18.5 (range: 0.0–60.0)	28.94 ± 22.5 (range: 0–72.0)
EQ‐5D	0.27 ± 0.35 (range: −0.6‐0.8)	0.7 ± 0.27 (range: −0.2‐1.0)	0.54 ± 0.35 (range: −0.1‐1.0)

**TABLE 4:** Radiological parameters before, immediately after and six years after fusion surgery for the treatment of degenerative spondylolisthesis

Radiological parameters	Preoperative	Postoperative	6‐year follow‐up
Gliding angle (°)	9.7 ± 3.6 (range: 3.3–15.8)	11.5 ± 9.3 (range: 2.4–43.5)	14.7 ± 11.1 (range: 4.1–48.2)
Sacral inclination (°)	43.1 ± 10.7 (range: 14.1–66.9)	39.7 ± 10.0 (range: 8.7–55.2)	37.1 ± 7.4 (range: 22.6–54.0)
Anterior displacement (mm)	25.1 ± 24.2 (range: 3.1–111.0)	12.5 ± 6.2 (range: 4.1–23.1)	21.8 ± 9.0 (range: 8.0–41.1)
Sagittal rotation (°)	71.4 ± 4.6 (range: 63.1–81.3)	72.2 ± 5.5 (range: 64.1–83.4)	75.8 ± 11.5 (range: 54.2–91.3)
Lumbar lordosis (°)	46.2 ± 15.7 (range: 14.5–81.4)	40.8 ± 11.8 (range: 17.0–63.2)	52.4 ± 11.5 (range: 26.4–69.2)
Sacral slope (°)	38.1 ± 9.6 (range: 18.2–53.1)	38.3 ± 5.9 (range: 28.7–53.2)	39.3 ± 6.8 (range: 27.2–52.1)
Pelvic tilt (°)	24.0 ± 6.3 (range: 12.5–33.9)	23.9 ± 4.4 (range: 15.6–29.7)	25.1 ± 9.0 (range: 8.4–41.2)
Pelvic incidence (°)	61.8 ± 10.6 (range: 46.2–83.3)	58.6 ± 7.1 (range: 41.4–67.7)	64.3 ± 12.2 (range: 42.9–87.3)

### Radiological Outcome

In terms of the radiological outcome the 6‐year follow‐up data showed a mean sacral inclination of 37.1° ± 7.4 (range: 22.6–54.0), a mean sacral slope of 39.3° ± 6.8 (range: 27.2–52.1) and a mean pelvic incidence of 64.3° ± 12.2 (range: 42.9–87.3).

The sacral slope and the sacral inclination correlated clearly (*r*_s_ = 0.637) as well as the sacral slope and the pelvic incidence (*r*_*s*_ = 0.500), defining both correlations as statistically significant with a *P* < 0.05. The pelvic incidence also strongly correlated with the pelvic tilt (*r*_s_ = 0.709, *P* < 0.01). Beyond that, only moderate and weak correlations were found (Table 1).

The remaining statistics of the clinical and radiological parameters are shown in Tables 3 and 4. The different radiological outcome parameters correlate with the clinical outcome measurements as described above. Inter‐ and intraclass reliability was excellent, and the ICC for the sagittal radiological parameters was between 0.887 and 0.956. The radiological data for all 17 patients is shown with mean values, standard deviation, and the minimum to maximum range at three time points: preoperatively, postoperatively and at time of 6 years of follow‐up.

## Discussion

### Long‐term Outcomes after Lumbar Fusion

This study shows that posterior and transforaminal lumbar interbody fusion are sufficient treatment techniques to address vertebral slippage. Additionally, these techniques facilitate bony fusion and lead to satisfactory long‐term results due to surgical realignment. Regarding the relation of clinical outcome and radiological parameters 6 years after fusion surgery the pre‐ and postoperative change of the sagittal rotation and the pelvic tilt were found to affect the clinical outcomes the most. This is demonstrated by the negative correlation of the change in sagittal rotation and ODI scores as well as the negative correlation of the change of the pelvic tilt and the EQ‐5D scores. A high ODI score is related to a more severe disability due to the patient's pain, mobility, and quality of life. Restoring the alignment of the malrotated vertebra by either the PLIF‐ or TLIF‐technique and achieving the correct position of the slipped vertebra is, according to this study, clearly preferable especially in comparison to alternative techniques like *in situ* spondylodesis or decompression only. Several studies support that conclusion, recommending a reduction of the displaced vertebra and fusion of the affected segment.^26^, ^27^, ^28^

**Fig. 2:**
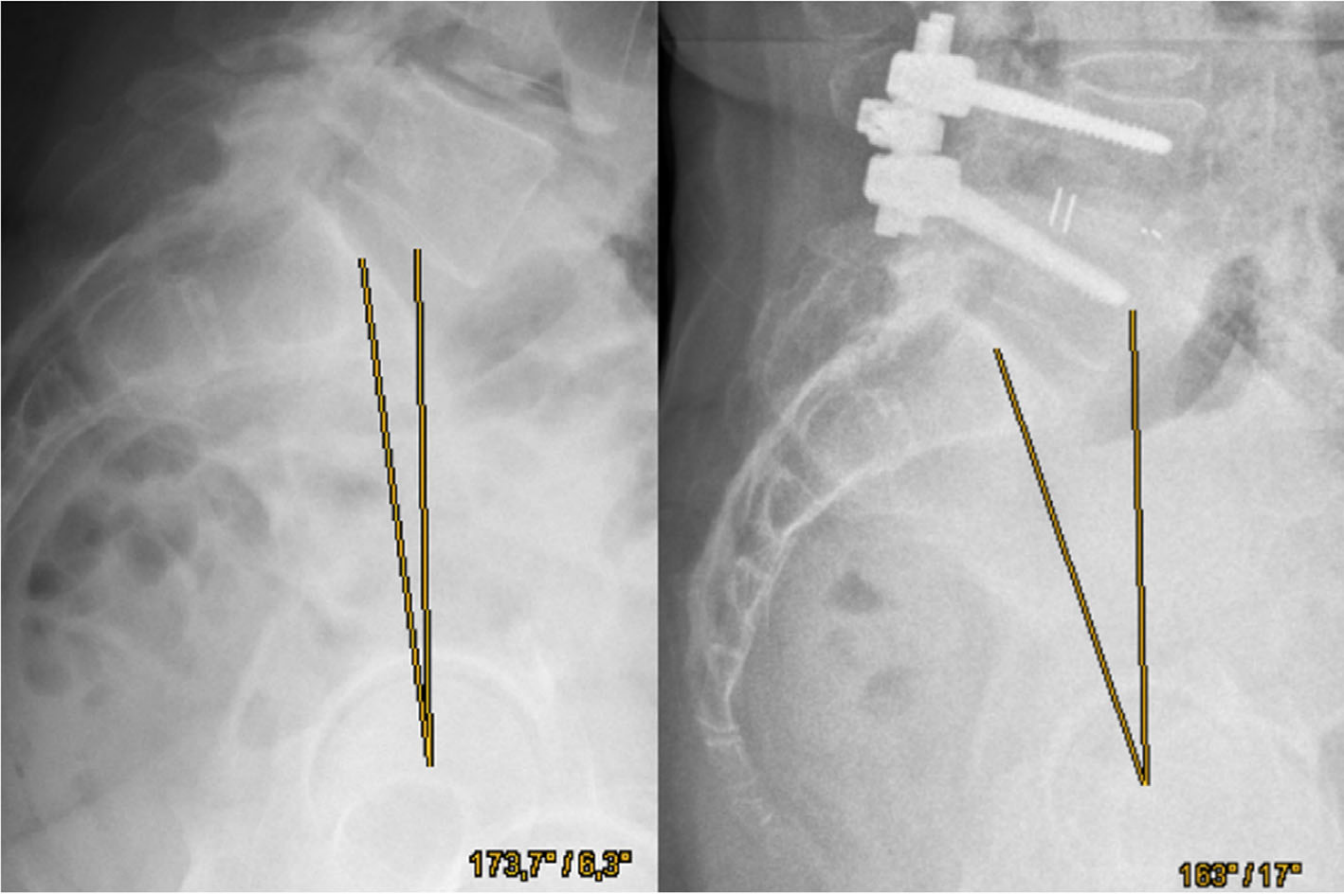
Pre‐ and postoperative (PLIF L4/5) lateral standing radiographs of the lumbar spine. The pelvic tilt is defined as the angle between the two depicted lines. The first line goes from the middle of the superior endplate of S1 to the middle of the femoral head and the second line is the vertical reference. The greater the difference of the pelvic tilt after fusion surgery compared to the preoperative value, the worse is the outcome after 6‐years when quantified by the EQ‐5D questionnaire.

Moreover, the results presented above show that an extensive change of the pelvic tilt is significantly associated with worse EQ‐5D scores, underlining that after repositioning the anteriorly displaced vertebra without changing the pelvic tilt, a better clinical outcome can be expected. Hence, the pelvic tilt should not be modified extensively or be addressed primarily by the surgery. The pelvic tilt should be changed as little as reasonably achievable according to our data (Fig. 2).

### The Impact of the Sagittal Alignment on the Postoperative Course after Fusion Surgery

The data presented are not only supported by Le Huec *et al.*, who describe the pelvic tilt as one important mechanism to compensate possible imbalance, but also by Lazennec *et al*. They identified an increase in pelvic tilt postoperatively to be associated with persistent pain following lumbar fusion surgery.^29^, ^30^ In accordance with our findings, Kim *et al*. describe in their retrospective study, that an improvement of the pelvic tilt following PLIF correlates with better clinical outcome scores using VAS and ODI in the treatment of patients suffering from degenerative spondylolisthesis.^31^ Other authors could not find a significant improvement of the clinical outcome according to a balanced pelvis after reduction surgery of lumbar spondylolisthesis.^32^, ^33^, ^34^, ^35^, ^36^, ^37^ Patients in pain due to fixed sagittal imbalance after spinal fusion are among other radiological parameters associated with an increased pelvic tilt postoperatively.^38^

Previously published data of the same registry this study is referring to found that one year after surgery the increase of sagittal rotation correlated with a better clinical outcome as measured by COMI, ODI, and VAS. Two years after surgery the clinical outcomes also improved significantly, but the three‐year data showed that the sagittal rotation did not correlate with the clinical outcome at that point of time.^39^ The 3‐year follow‐up data did, however, show a correlation between the sacral slope and correlation of the sacral inclination and the COMI score. This 6‐year follow‐up did not show similar results regarding the correlation of the COMI and the sacral slope and the sacral inclination. Instead, a significant correlation of the sacral slope and the sacral inclination (r*0.696) was found. Furthermore, the anterior displacement shows significantly negative correlation with the sacral inclination (r*−0.542) and the lumbar lordosis (r*0.632). This confirms the benefit of a restored lumbar lordosis after repositioning the slipped vertebra with fusion surgery. Targeted modulation of sagittal alignment parameters with dorsal lumbar fusion surgery is also described in other studies as a desirable technique.^40^, ^41^ This is one reason why Le Huec *et al*. recommend comparing sagittal alignment parameters of a group of patients with an asymptomatic population instead of referring to the mean values of the patients.^29^ According to their findings, many studies assume heterogenous standard values leading to a lower impact and comparability of the study. Nevertheless, restoring the physiological alignment of the spine, especially of the lumbar lordosis, can prevent poor clinical outcome and adjacent segment degeneration.^42^, ^43^

When trying to explain the disparities of the studies mentioned above, not only the different study designs, patients, methods, and surgical techniques affect the results and associated recommendations for surgical treatment. Funao *et al*. demonstrated that depending on the individual spinopelvic alignment, patients have different mechanisms and preconditions to compensate the slippage of vertebrae, and the change of spinopelvic alignment after surgery.^44^ These compensatory mechanisms represent additional individual factors, that are difficult to calculate before fusion surgery.^45^ Anyhow, according to the presented 6‐year data, restoring sagittal alignment as part of the repositioning of the anteriorly slipped vertebra should be suggested to improve the clinical long‐term outcome.

### Limitations and Future Considerations

This study has several limitations. Despite the high value of our long‐term follow‐up data, it is limited with regard to the number of patients included. Furthermore, generating a control group for the radiological parameters would have been desirable but is difficult to realize. Additionally, the inclusion of 17 patients only due to the low response rate limits the conclusions that were drawn based on the collected data.

Nevertheless, in accordance with the aims of this study, certain radiological parameters that correlated significantly with a desirable clinical outcome 6 years after lumbar fusion surgery were identified. Extensive modification of the pelvic tilt leads to inferior clinical outcomes as well as the insufficient reduction of the sagittal rotation angle of the displaced vertebra. Analyzing and understanding the sagittal balance of the spine when planning a surgical intervention is inevitable as described by Le Huec *et al*.^46^

In the future, it should be analyzed which fusion technique is best for which severity of DS. Further research is needed to identify the association of the above mentioned long‐term radiological alignment parameters and the clinical outcome clearly.

## Conclusion

Reducing the sagittal rotation as well as retaining the pelvic tilt were found to be the most important modifications of radiological alignment parameters that correlate with an improved long‐term clinical outcome six years after surgery for patients suffering from degenerative spondylolisthesis and treated with PLIF and TLIF. The preoperative measurement, understanding and interpretation of the sagittal spinal alignment parameters is essential for the treatment of degenerative spondylolisthesis.

## Author Contributions

Conceptualization, J.B. and T.P.; methodology, S.O., C.M.; software, S.O. and C.K.; validation, J.B., C.H., and T.P.; formal analysis, C.K. and V.H.; investigation, C.K. and N.K.; resources, P.E.; data curation, S.O., T.P., N.K.; writing ‐ original draft preparation, J.B. and T.P.; writing ‐ review and editing, J.B., C.M., T.P., C.H., and V.H.; visualization, T.P.; supervision, J.B. and P.E.; project administration, J.B. and T.P.; funding acquisition, not applicable. All authors read and agreed to the published version of the manuscript.

## Data Availability

All data is available, as a part of the SpineTango‐Registry.
